# Feasibility of a tailored home-based exercise intervention during neoadjuvant chemotherapy in breast cancer patients

**DOI:** 10.1186/s13102-022-00420-6

**Published:** 2022-02-25

**Authors:** Kathleen M. Sturgeon, Amanda M. Smith, Elizabeth H. Federici, Namratha Kodali, Renée Kessler, Edward Wyluda, Leah V. Cream, Bonnie Ky, Kathryn H. Schmitz

**Affiliations:** 1 Department of Public Health Sciences, Penn State College of Medicine, Hershey, PA USA; 2 School of Medicine, University of Pennsylvania, Philadelphia, PA USA; 3 Department of Hematology/Oncology, Penn State College of Medicine, Hershey, PA USA; 4 Penn State Health Medical Group, Andrews Patel Hematology/Oncology, Harrisburg, PA USA; 5 Department of Public Health Sciences, Milton S. Hershey Medical Center, Penn State College of Medicine, Penn State Cancer Institute, 500 University Drive, mail code CH69, Hershey, PA 17033 USA

**Keywords:** Aerobic exercise, Home-based, Drug therapy, Quality of life, Fitness

## Abstract

**Purpose:**

To evaluate the feasibility of a home-based moderate-to-vigorous intensity, phased (introduction, intermediate, maintenance), exercise prescription in breast cancer patients receiving cardiotoxic neoadjuvant chemotherapy.

**Methods:**

Nineteen breast cancer patients were randomized to intervention or control for the duration of chemotherapy (16–24 weeks). The intervention was one aerobic exercise session at 80–90% VO_2max_ for 25 min/week and 65%-75% VO_2max_ for ≥ 50 min/week. Adherence to the tailored home-based program was assessed by heart rate monitors. Acceptability, tolerability, feasibility, efficacy, change in VO_2max_, and patient reported outcomes, safety, and clinical events were assessed.

**Results:**

25.7% of eligible women consented (acceptability). Adherence was 87.6%. Women were not able to maintain exercise intensity as chemotherapy progressed (23.7% of exercise minutes were completed at prescribed heart rate during maintenance). Efficacy of the intervention was demonstrated by maintenance of VO_2max_ (−1.0 ± 13.2%) compared to (−27.5 ± 7.4%) the control group. Further, during and after therapy, patients in the intervention arm reported less fatigue (control-baseline: 14.4 ± 15.9; midpoint: 19.0 ± 11.4; follow-up: 29.4 ± 20.0; intervention-baseline: 29.2 ± 24.6; midpoint: 24.6 ± 14.4; follow-up: 23.6 ± 11.9), impairment in activities (control-baseline: 13.7 ± 16.0; midpoint: 32.8 ± 17.0; follow-up: 58.6 ± 27.9; intervention-baseline: 38.7 ± 31.8; midpoint: 47.1 ± 27.5; follow-up: 47.5 ± 31.0), and pain (control-baseline: 80.8 ± 17.1; midpoint: 73.9 ± 20.7; follow-up: 50.7 ± 25.7; intervention-baseline: 68.7 ± 28.4; midpoint: 61.4 ± 22.5; follow-up: 65.3 ± 22.4). There were no differences in adverse events, treatment delays, or pathological complete response.

**Conclusions:**

Neoadjuvant breast cancer patients maintained approximately one hour/week of moderate-intensity exercise over the course of their treatment. Further, this volume of exercise was sufficient to maintain fitness capacity and quality of life compared to the control group.

*Trial registry*: ClinicalTrials.gov Identifier: NCT03280836, prospectively registered 9/13/2017, https://clinicaltrials.gov/ct2/show/NCT03280836.

## Background

Breast cancer continues to be the leading site of new cancer cases for women in the United States. It is estimated that in 2019, 268,600 women were diagnosed with breast cancer [1]. The trend in annual rates of new breast cancer cases has remained largely unchanged in the last 20 years. Uptake in screening practices and improved treatment have contributed to an increasing pool of breast cancer survivors. While 5-year cancer-specific survival rates in early-stage breast cancer patients are 90–100%, [2] patients diagnosed with locally advanced breast cancer, or, breast cancer patients with an aggressive tumor subtype, have worse outcomes. Five-year disease-free survival rates for locally advanced, [3] ER+/PR+/HER2− or HER2+, [4] and TNBC [4] are 85%, 78%, and 69% respectively. These statistics highlight that (1) breast cancer continues to affect many women in the United States, (2) this trend remains unchanged, (3) the number of breast cancer survivors is increasing, and (4) there is room for improvement for patients with worse prognostic indicators. Additionally, side effects of cancer treatment, such as cardiotoxicity, may predispose breast cancer patients to increased risk of mortality from cardiovascular disease compared to the general United States population [5].

Our pilot study used exercise as a non-pharmacological intervention to improve treatment-related outcomes in breast cancer patients with worse prognostic indicators. Neoadjuvant chemotherapy is a standard of breast cancer clinical care for several tumor types: locally advanced tumors, HER2+ tumors, and TNBC [6]. These tumor types also have the poorest relapse-free survival rates. Pre-clinical work demonstrates exercise-induced improvement in tumor response to chemotherapy [7]. We and others have reported exercise-induced improvements in tumor perfusion leading to greater efficacy of chemotherapy in mice [7, 8]. However, it is unknown if exercise clinically reduces tumor burden or improves therapeutic efficacy. There is also uncertainty as to the dose of exercise appropriate for hypothesized exercise-induced improvements in therapeutic efficacy in humans.

The dose of exercise required to improve therapeutic efficacy may differ from the dose of exercise required to protect the heart against cardiotoxic chemotherapies. Indeed, we observed that while a low-to-moderate level of exercise failed to mitigate cardiac remodeling due to doxorubicin in tumor bearing animals, [9] this low-to-moderate level of exercise in mice did improve therapeutic efficacy [7, 9]. Based on previous work in cardiology, the threshold dose of exercise for cardiac benefit may be found at higher intensities [10]. Yet, moderate-to-vigorous intensity exercise may not be feasible in breast cancer patients actively receiving chemotherapy. The 2019 American College of Sports Medicine (ACSM) expert panel on exercise in cancer highlighted that an individual’s response to a given exercise stimulus may vary due to the direct effects of cancer treatments on physiological systems (e.g., anemia), side effects of cancer treatment (e.g., cancer-related fatigue may lower exercise tolerance), or demographics factors (e.g., age) [11, 12]. Furthermore, during active treatment an individual’s ability to tolerate exercise may fluctuate from day to day or week to week.

Designing exercise oncology interventions that work for breast cancer patients is important because cancer patients tend to present at diagnosis with lower fitness capacity (− 17%) compared to healthy, sedentary, similar aged, women [13]. Fitness capacity becomes further impaired (− 10%) following cancer treatment [13]. This decline is sustained for years after treatment compared to age-matched controls [14]. These findings are of key importance given that even small differences in fitness capacity (1 MET or 3.5 mL/kg/min) are associated with a significantly higher risk for cardiovascular mortality (18%) [15, 16]. Therefore, an intervention during neoadjuvant chemotherapy to maintain fitness capacity and mitigate declines in fitness capacity is a clinically meaningful approach to decrease risk of overall and cardiovascular specific mortality in breast cancer patients [5, 16–18].

While exercise is a meaningful complementary approach during cancer treatment, it may also be an overwhelming addition to already significant time and resource burdens on neoadjuvant breast cancer patients. Appointments at hematology/oncology, phlebotomy, and the infusion suite are often interspersed with planning appointments with surgical oncology, plastics and reconstructive surgery, and radiation oncology. Thus, home-based exercise interventions, rather than supervised programs, have fewer logistical barriers for uptake [12]. In addition, while it is known supervised exercise is more efficacious, there is still biological benefit to home-based exercise [11]. Strong evidence supports the benefits of exercise oncology during cancer treatment for decreased fatigue, improved health related quality of life, and increased physical function [11]. Yet, there is currently insufficient evidence regarding the benefit of exercise oncology for outcomes such as pain, work productivity, and treatment tolerance [11].

Ongoing work in our laboratory and others utilizes pre-clinical models to determine optimal dosing strategies for exercise oncology to achieve specific outcomes (therapeutic efficacy or cardiotoxicity) [7–9]. In order to translate results to the clinical setting we conducted a multi-center pilot study to examine the feasibility of a moderate-to-vigorous, home-based, remotely delivered, exercise program in breast cancer patients who were initiating neoadjuvant chemotherapy. We hypothesized that a tailored, phased, moderate-to-vigorous intensity, exercise prescription conducted from first to final chemotherapy infusion, and weekly tele-coaching would be acceptable, tolerable, feasible, and efficacious in mitigating treatment-related side effects.

## Methods

### Sample population and recruitment

Nineteen female non-metastatic breast cancer patients who were scheduled to receive neoadjuvant chemotherapy were enrolled across three sites between 2017 and 2020. Identification of eligible patients occurred at the Penn State Cancer Institute (PSCI, Hershey, PA), Andrews & Patel community oncology practice (A&P, Harrisburg, PA), and the University of Pennsylvania (UPenn, Abramson Cancer Center, Philadelphia PA). The study was approved by the Penn State College of Medicine Institutional Review Board and written informed consent was obtained prior to any study procedures. Inclusion criteria included: Breast cancer stage I–III, English speaking patients, > 18 years with documented breast cancer for whom treatment with cardiotoxic chemotherapy regimens was planned (Taxotere, Carboplatin, Herceptin + Perjeta; TCH + P, or, Adriamycin, cyclophosphamide, Taxol; ACT). Exclusion criteria included pregnancy, presence of heart disease, or previous history of anthracycline chemotherapy. Approval to approach the patient was approved by the primary medical oncologist. Additional eligibility criteria was confirmed by interview prior to consent (absence of heart disease, no contraindications for exercise testing or training, and sedentary defined as < 75 min/wk of self-reported moderate intensity leisure-time physical activity over the past month). Acceptability was defined as the consent rate amongst eligible patients.

The study was powered at 80% to detect at 4 ml/kg/min difference in VO_2max_ between control and intervention groups (n = 20) at an alpha level of 0.05 [19]. Four ml/kg/min is ~ equivalent to a 1-MET difference, which has been associated with a 23% reduction in risk for cardiovascular events in women [20]. The study was closed to recruitment at n = 19 due to the COVID-19 pandemic (resulting in 78% power).

### Primary endpoint

For safety reasons, submaximal fitness testing was conducted with 10-lead ECG cardiac monitoring with expired gas analysis (ParvoMedics TrueOne® 2400, Sandy UT) [21]. Patients were asked to complete a modified Bruce Protocol to volitional fatigue or through the protocol stage where 80% heart rate maximum (HR_max_) was reached. The same measurement technicians conducted baseline and follow up fitness testing. Blood pressure was measured and rating of perceived exertion (RPE) on the modified Borg Scale was evaluated two minutes into each protocol stage. Testing was conducted before starting chemotherapy and in the window following chemotherapy but prior to surgical resection. VO_2max_ was predicted at age-adjusted HR_max_ using the Tanaka formula and the individual linear slope generated from graphing VO_2_ and HR from the submaximal test on their respective graphical axis [22, 23].

### Measurements

Following baseline VO_2max_ testing patients were randomized 1:1 using an a priori computer-generated sequence. Patients engaged in an exercise education session before they received intervention materials on exercise training. The education session provided instruction regarding proper warm-up, cool-down, stretches, proper footwear for injury prevention, and understanding RPE [24]. For women randomized to the intervention arm, this education session took place at their baseline exercise testing session. For women randomized to the control arm, this education session took place at their follow up exercise testing session.

Surveys and questionnaires were also collected at baseline, midpoint, and follow up. Midpoint was week 8 or 9 depending on chemotherapy regimen. Patients completed the Godin Physical Activity Questionnaire, Work Productivity and Activity Impairment Questionnaire (WPAI), EuroQol 5D (EQ-5D), RAND 36-Item Short Form (SF-36), and the Multidimensional Fatigue Symptom Inventory (MFSI-SF) short form, and an adverse events survey [25–28]. The Leisure Score Index from the Godin Physical Activity Questionnaire was calculated as previously described [25]. Additionally, at follow up, patients completed an injury history questionnaire to detect any exercise-related events [29]. Throughout the intervention, electronic medical records and personal communications with patients were monitored for deviations from normal health (adverse events) and documented. Electronic medical records were monitored for changes to treatment schedule and synoptic pathology reports were abstracted following surgical resection.

### Control

Participants randomized to the control group were asked to maintain their usual level of physical activity and to not engage in any new exercise program during study participation. Participants in the control group were given the exercise DVD, exercise binder, and exercise prescription based on their follow up exercise test, following completion of all study visits (including their exercise safety education session), and clearance from surgical oncology.

### Intervention

Patients randomized to the intervention group received three commercially available aerobics DVDs and an informational binder of aerobic exercises. Patients were instructed to self-select the combination of activities that places them in their appropriate heart rate zone. Patients were coached by the exercise interventionist on this during their education session and throughout the intervention. Phone calls with the coach were conducted 1x/week, and typically lasted 10–20 min. All participants interacted with the same exercise interventionist and calls revolved around discussions of: review of weekly progress from HR monitors and self-report logs, HR and duration of exercise goals for the next week, discovery of daily activities/time use to identify strategies and opportunities to address challenges or barriers to exercise, general side effects of chemotherapy treatment, and adaptation of the exercise program to the course of side effects and daily life.

The DVDs and binder included exercises ranging from continuous in-home walking routines (for rainy/cold days or those with limitations for outside activities) to higher intensity continuous aerobic activities such as step taps, skipping, and jumping jacks (with modifications/variations shown). The DVD titles were: START! Walking At Home® with Leslie Sansone; Just Walk, 5 Boosted Miles with Leslie Sansone; and 5 Mix & Match Miles with Jessica Smith. The movements in the DVDs were aerobic and combinations of: marching in place, side touches, step taps, knee lifts, front/back kicks, hamstring curls, walking forward and backward, jogging in place, skater hops, grapevine, arm swings, overhead reaches, clapping, front presses, and arm rows.

Patients wore a Polar Heart Rate monitor (US model RS400, Polar Electro Inc., Lake Success, NY) during exercise to monitor exercise intensity. The study interventionist reviewed data from the heart rate monitors to objectively monitor exercise adherence. Patients in the intervention group were also asked to keep an exercise log with the date, time, average heart rate obtained from a heart rate monitor, duration of workout and stretching, and any comments regarding the workout. Logs were also reviewed weekly by the interventionist and discussed on the weekly coaching call [30].

The exercise intervention was designed to target 75 min per week of aerobic activity at 70–90% of baseline VO_2max,_ or associated RPE. In Weeks 1–4 (Introductory Phase) of the program, the frequency, duration, and intensity of aerobic exercise was progressively increased from an initial prescription of 60 min/wk (intervention frequency was suggested at 3 sessions/wk, but allowed to be broken up into other increments for the entirety of the intervention) at 50% of VO_2max_ (RPE = 2), up to 75+ min/wk at 60% of VO_2max_ (RPE = 3–4) at the end of week 4. The goal of these sessions was to introduce aerobic exercise, including warm-up and proper form as well as integration with lifestyle.

In weeks 5–11 (Intermediate Phase), the goal was to increase exercise intensity from 60 to 80% VO_2max_ in a ramped fashion. Specifically, by week 11, the exercise prescription was 65–75% VO_2max_ (RPE = 5–6) for two sessions per week (or 2/3 s of weekly exercise time) and in the remaining session (or 1/3 of weekly exercise time), aerobic exercise intensity was 80% + VO_2max_ (RPE = 7–8). In weeks 12–24 (Maintenance Phase) participants were asked to maintain the exercise prescription from week 11. This tailored exercise prescription was based on initial VO_2max_ and delivered through personal heart rate zones. During the maintenance phase, if side effects of chemotherapy limited the ability of patients to reach their heart rate goal, they were encouraged to work at the RPE associated with the HR zone.

### Statistical analysis

Descriptive statistics were reported for baseline variables including percentages for categorical variables and means and standard deviations for continuous variables. Acceptability was defined by the proportion of approached patients whom consented to the study. Tolerability was defined (1) by the number of weekly exercise minutes completed, and (2) by the number of weekly exercise minutes completed at the prescribed exercise intensity according to the patient’s HR monitor. Self-reported exercise time in the patient log book was utilized in certain instances (malfunctioning watch, missed wear) to determine weekly exercise minutes only if 80% of their log entries could be validated against the heart rate monitor data. Feasibility was defined by the loss to follow up for final measurements (VO_2max_ and patient reported outcome questionnaires). In addition to these pilot study observations, the primary outcome, and efficacy of the intervention, was difference in fitness capacity between the intervention and control groups at the follow up exercise test [21]. Baseline adjusted linear regression was used to assess differences between groups at the follow up exercise test. A mixed-model repeated measures analysis of variance was used to compare between group differences over time for the secondary outcome survey variables. Fisher’s exact tests and χ^2^-tests were used to examine between group differences in categorical variables and significance level was set at *P* < 0.05.

## Results

**Fig. 1:**
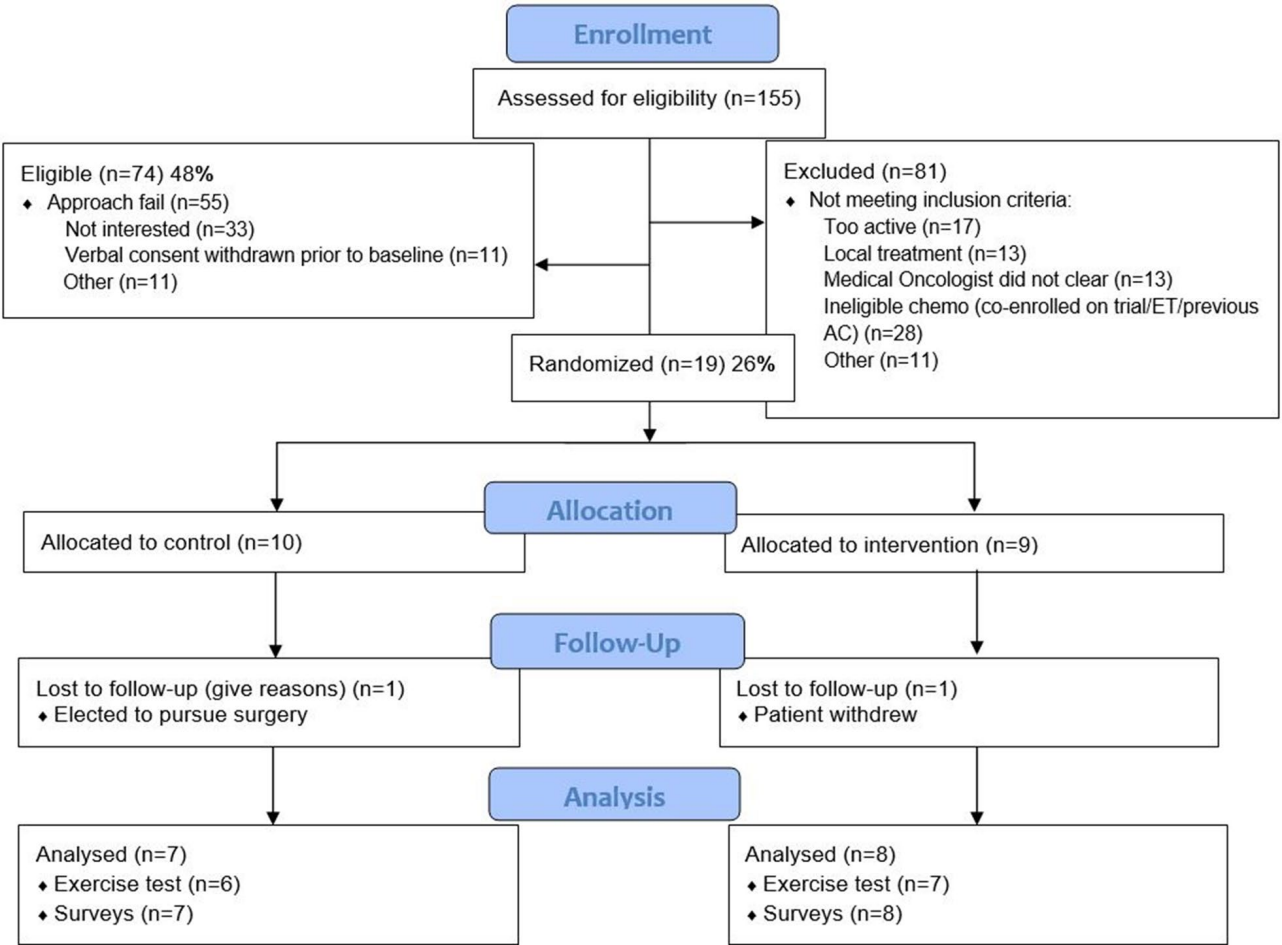
Study CONSORT diagram. Across three centers, 155 breast cancer patients scheduled to begin neoadjuvant chemotherapy were assessed for eligibility. Forty-eight percent were eligible for the study, and 19 women were enrolled. Of the 10 women in the control group, one withdrew after her first chemotherapy infusion and three had significant side effects to chemotherapy which resulted in loss to follow up for exercise testing. In the intervention group, one patient withdrew one week following randomization and another patient was lost to follow up for inability to schedule testing

**Fig. 2:**
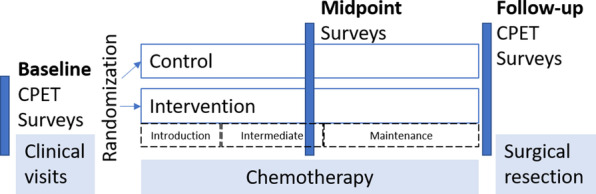
Study schema of research activities and neoadjuvant chemotherapy. All baseline testing (cardiopulmonary exercise test (CPET) and surveys) was conducted prior to starting chemotherapy and the exercise intervention was initiated concomitant to chemotherapy. Follow up testing was completed prior to surgical resection. Chemotherapy treatment lasted 16–24 weeks depending on treatment and individual delays. The introductory phase of the intervention lasted 4 weeks, followed by 7 weeks of an intermediate ramp phase. At week 12 the maintenance phase began and continued until chemotherapy and follow-up testing was completed

Figure 1 provides the CONSORT diagram for the trial. Patients were identified by screening schedules of breast oncologists for patients recommended for neoadjuvant chemotherapy. Overall, 155 women were assessed for eligibility (n = 77 UPenn, n = 61 PSCI, n = 17 A&P). The number of patients meeting certain exclusion criteria varied between sites. Specifically, co-enrollment on a clinical trial was only observed for patients at UPenn (n = 16) and 65% of the patients that failed eligibility screening due to high physical activity levels were screened at UPenn. Overall, our acceptability rate was 25.7% with 19 women consented of 74 eligible women that were approached. We observed that while we obtained verbal consent from 30 women (40.5%) that were eligible, there was a 15% withdraw rate of verbal consent due to being overwhelmed with their cancer diagnosis prior to starting chemotherapy, in addition to the burden of appointments, and altered daily life course. Two patients withdrew from the study within the first chemotherapy cycle. Feasibility for the primary outcome of VO_2max_ was 68% (10.5% withdrew, 5.3% loss to follow up, 15.8% chemotherapy-induced contraindications to exercise testing), and 79% completed final surveys (10.5% withdrew, 10.5% loss to follow up). Figure 2 displays the timeline of research activities compared to clinical care milestones (start/end of chemotherapy).

The study demographic ranged in age from 26 to 64 years and was 31% minority and 100% non-Hispanic (Table 1). At baseline, 84% of women were working full or part-time, and 53% had household incomes greater than $75,000. The majority of the women were coupled (74%), non- or ex-smokers (84%). Clinically, the majority of women had stage 2 breast cancer and were HER2+ (74%). There were no significant differences in distribution of demographic or clinical characteristics between the intervention and control groups (Table 1).

**Table 1:** Demographic and clinical characteristics

	Overall n = 19	Control n = 10	Intervention n = 9	*P*-value
Age (years)	49.4 ± 10.5	51.5 ± 9.5	47.0 ± 11.7	0.37
Race n, (%)				
White	13 (69%)	8 (80%)	5 (56%)	0.40
Black	5 (26%)	2 (20%)	3 (33%)	
Other	1 (5%)	0 (0%)	1 (11%)	
Ethnicity n, (%)				
Not hispanic	19 (100%)	10 (100%)	9 (100%)	
Working status n, (%)				
Full time (at least 1 job)	12 (63%)	7 (70%)	5 (56%)	0.73
Part time (no FT)	4 (21%)	2 (20%)	2 (22%)	
Other	3 (16%)	1 (10%)	2 (22%)	
Household income n, (%)				
0–49,999	5 (26%)	3 (30%)	2 (22%)	0.18
50,000–75,000	4 (21%)	3 (30%)	1 (11%)	
75,001–125,000	6 (32%)	1 (10%)	5 (56%)	
125,000+	4 (21%)	3 (30%)	1 (11%)	
Education n, (%)				
HS/some college	4 (21%)	2 (20%)	2 (22%)	0.74
Associates/Bachelors	8 (42%)	5 (50%)	3 (33%)	
Graduate school	7 (37%)	3 (30%)	4 (44%)	
Partnered n, (%)				
Not partnered	5 (26%)	4 (40%)	1 (11%)	0.15
Coupled	14 (74%)	6 (60%)	8 (89%)	
Smoking status n, (%)				
Non or ex-smoker	16 (84%)	9 (90%)	7 (78%)	0.53
Current smoker	3 (16%)	1 (10%)	2 (22%)	
Site n, (%)				
CCC	6 (32%)	3 (30%)	3 (33%)	0.97
CI	9 (47%)	5 (50%)	4 (44%)	
Community	4 (21%)	2 (20%)	2 (22%)	
Stage n, (%)				
1	4 (21%)	2 (20%)	2 (22%)	0.93
2	10 (53%)	5 (50%)	5 (55%)	
3	5 (26%)	3 (30%)	2 (22%)	
Tumor type n, (%)				
HR+ HER2+	6 (32%)	4 (40%)	2 (22%)	0.81
HR+ HER2−	2 (11%)	1 (10%)	1 (11%)	
HR-HER2+	8 (42%)	4 (40%)	4 (44%)	
HR-HER2−	3 (16%)	1 (10%)	2 (22%)	
Chemo n, (%)				
ACT	5 (26%)	2 (20%)	3 (33%)	0.51
TCHP	9 (47%)	6 (60%)	3 (33%)	
ACTH	5 (26%)	2 (20%)	3 (33%)	

Data presented as mean ± SD for continuous data and n (%) for categorical data. Abbreviations include: Comprehensive Cancer Center (CCC), Cancer Institute (CI), hormone receptor (HR), human epidermal growth factor receptor 2 (HER2), ACT denotes Adriamycin® (doxorubicin), cyclophosphamide, and Taxol® (paclitaxel), TCHP denotes Taxotere® (docetaxel), carboplatin, Herceptin® (trastuzumab), Perjeta® (pertuzumab)

Of the nine patients in the exercise intervention group, one withdrew prior to starting, and two did not participate past week two. Of the six women that engaged in the exercise training, we observed that it was very difficult for patients to maintain the prescribed exercise intensity during the maintenance phase of the exercise program (Table 2). However, they were able to maintain a schedule of over an hour of exercise per week. While not all of that time exercising was spent at the prescribed heart rate (exercise intensity), it was over 85% of the prescribed exercise duration. The observed heart rates were on average consistent with exercising at 60–65% of VO_2max_ for the entire intervention.

**Table 2:** Tolerability of a ramped, moderate-to-vigorous intensity, exercise prescription with weekly tele-coaching

	Exercise time (min/wk)	Average HR during exercise (bpm)	Average HR prescribed (bpm)	Exercise time at Rx HR (%)
Introduction phase	64.2 ± 6.8	134.2 ± 9.5	122.7 ± 13.3	72.7 ± 11.5
Intermediate phase	67.1 ± 5.7	139.3 ± 3.5	141.9 ± 12.3	61.0 ± 5.0
Maintenance phase	66.0 ± 10.4	137.8 ± 7.3	149.0 ± 11.5	23.7 ± 7.1

Mean ± SEM for patients that progressed at least 2 weeks into the introduction phase (n = 6)

**Fig. 3:**
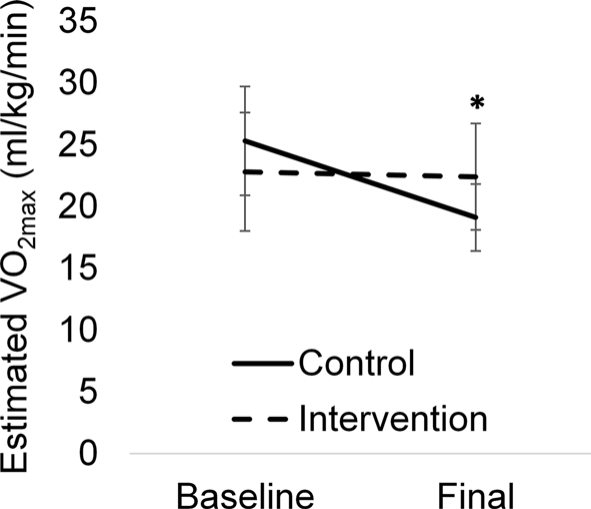
Fitness capacity prior to and following neoadjuvant chemotherapy in breast cancer patients. VO_2max_ estimated from a submaximal exercise test is presented for the control (black line) and intervention (dashed line) groups at baseline and follow up. Mean ± SD. *P = 0.04

**Fig. 4:**
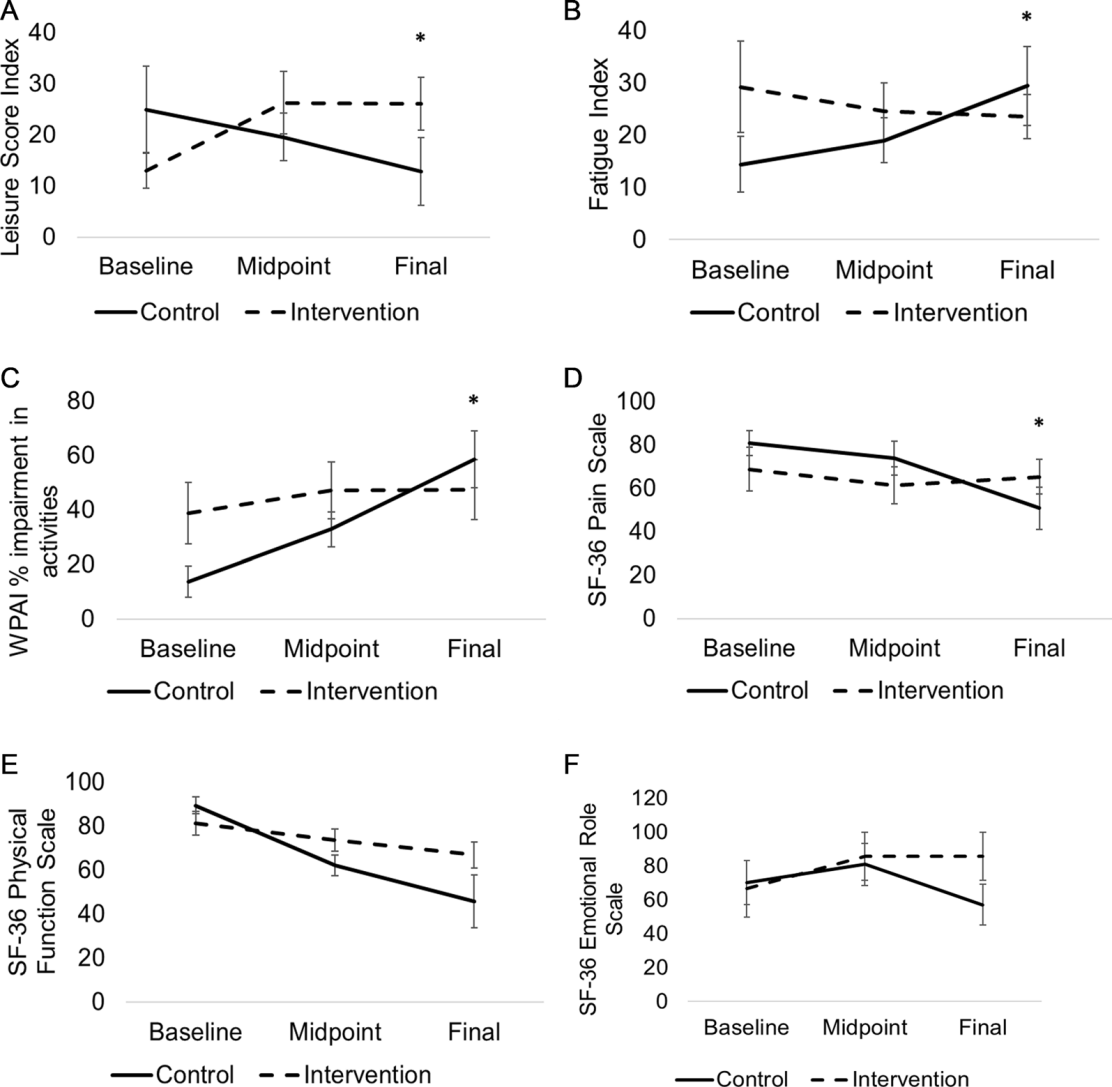
Quality of life prior to, during, and following neoadjuvant chemotherapy in breast cancer patients. Leisure score index measured with the Godin Physical Activity Questionnaire (**A**), *P* = 0.03, Fatigue Index from the MFSI-SF (**B**), *P* = 0.01, impairment in regular daily activities measured with the Work Productivity and Activity Impairment (WPAI) Questionnaire (**C**), *P* = 0.02, and several scales derived from the RAND 36-Item Short Form (SF-36) such as pain (**D**), *P* = 0.02, physical function (**E**), *P* = 0.06, and role of emotions in daily life (**F**), *P* = 0.09, are presented for the control (black line) and intervention (dashed line) groups at baseline, midpoint, and follow up. Mean ± std. error mean. *P < 0.05

The intervention was sufficient in maintaining fitness capacity (baseline: 19.5 ± 5.9 ml/kg/min; follow up: 18.9 ± 5.8 ml/kg/min), whereas patients in the control group saw a significant decrease (baseline: 18.5 ± 3.7 ml/kg/min; follow up: 14.9 ± 3.0 ml/kg/min) in their fitness capacity (Fig. 3). Estimated VO_2max_ decreased from baseline by −24.5% in the control group and by −1.7% in the intervention group. The exercise intervention significantly increased self-reported leisure time physical activity (Fig. 4A) (control-baseline: 24.9 ± 25.7; midpoint: 19.6 ± 12.5; follow up: 12.8 ± 17.5; intervention-baseline: 13.0 ± 8 0.6; midpoint: 26.3 ± 16.1; follow up: 26.0 ± 14.6), while also significantly mitigating increases in fatigue (control-baseline: 14.4 ± 15.9; midpoint: 19.0 ± 11.4; follow up: 29.4 ± 20.0; intervention-baseline: 29.2 ± 24.6; midpoint: 24.6 ± 14.4; follow up: 23.6 ± 11.9), and impairment of daily activities (control-baseline: 13.7 ± 16.0; midpoint: 32.8 ± 17.0; follow up: 58.6 ± 27.9; intervention-baseline: 38.7 ± 31.8; midpoint: 47.1 ± 27.5; follow up: 47.5 ± 31.0), observed in the control group (Fig. 4B, C). Higher scores on the SF-36 survey indicate more favorable outcomes. We observed that the intervention group had less pain (Fig. 4D) (control-baseline: 80.8 ± 17.1; midpoint: 73.9 ± 20.7; follow up: 50.7 ± 25.7; intervention-baseline: 68.7 ± 28.4; midpoint: 61.4 ± 22.5; follow up: 65.3 ± 22.4), better physical function (*P* = 0.06, Fig. 4E) (control-baseline: 89.4 ± 11.3; midpoint: 62.1 ± 12.2; follow up: 45.7 ± 31.5; intervention-baseline: 81.2 ± 15.5; midpoint: 73.6 ± 13.1; follow up: 66.9 ± 16.7), and less limitation in their role due to emotional limitations (*P* = 0.09, Fig. 4F) (control-baseline: 70.4 ± 38.9; midpoint: 80.9 ± 32.5; follow up: 57.1 ± 31.7; intervention-baseline: 66.7 ± 47.1; midpoint: 85.7 ± 37.8; follow up: 85.7 ± 37.7). Lastly, there were no significant differences in exercise adverse events (control: 0.9 ± 0.9; intervention: 0.7 ± 0.8), treatment delays (control: 55%; intervention: 37%), or pathological complete response (control: 67%; intervention: 75%) between groups.

## Discussion

We conducted a multi-center pilot study to examine the acceptability, tolerability, feasibility, and efficacy of a moderate-to-vigorous, home-based, remotely delivered, exercise program in breast cancer patients beginning neoadjuvant chemotherapy. We hypothesized that an individualized, phased, moderate-to-vigorous intensity, exercise prescription and weekly tele-coaching would be tolerable and improve fitness capacity. We observed that our exercise intervention maintained, but did not increase, fitness capacity in women receiving neoadjuvant chemotherapy. Women in the exercise intervention group maintained, on average, a moderate, but not vigorous, exercise intensity over the course of the exercise intervention. Further, while the flexibility built into our home-based, remotely delivered, individualized, exercise program was successful in keeping women adherent to the time component of our exercise dose, we observed limited tolerability of our exercise program for the intensity component of our exercise dose.

Our results indicate that approximately 65 min per week of moderate intensity aerobic exercise was sufficient to maintain fitness capacity. Similar to previous studies specifically conducted in breast cancer patients during cancer treatment, we observed that adherence to exercise training is high when the exercise prescription is flexible within a given set of a priori guidelines [31, 32]. Also, similar to previous work, high intensity aerobic exercise is difficult to maintain for breast cancer patients as chemotherapy cycles progress [33, 34]. Home based aerobic exercise interventions in breast cancer patients during chemotherapy (40–150 min/wk, moderate intensity, weekly calls, heart rate monitored) have been successful in maintaining or improving fitness capacity as measured by a 6-min walk test, [35, 36] and have improved fatigue levels [36, 37]. However, unlike these studies, our intervention was conducted for the entire length of chemotherapy (16–24 weeks) and not limited to 8, or 12 weeks [21, 38].

Disruptive world events such as the COVID-19 pandemic often spur medical innovation. Mobile health and remote communication technologies have become significantly more pervasive because of the pandemic. While the majority of our study was conducted prior to vast numbers of the population becoming comfortable with video chatting, we believe our home-based intervention would be conducive to an online lifestyle program. Indeed, the rapport created between interventionist and participant during such a challenging time as chemotherapy treatment would only be enhanced by video, rather than phone, conversations. Such an adaptation also could allow for synchronous coaching, to parallel, supervised interactions.

It is well established that fitness capacity, and change (increase or decrease) in fitness capacity, are powerful predictors of mortality in healthy adults as well as those with cardiovascular disease, even after controlling for traditional cardiovascular risk factors [16–18]. In a meta-analysis specific to breast cancer survivors, it was observed that the fitness capacity (VO_2max_) of a 50 year old breast cancer survivor was most similar to that of a sedentary 60 year old woman [13]. Thus, exercise prescriptions during active treatment to mitigate declines in fitness capacity is clinically meaningful with regard to decreasing risk of overall and cardiovascular specific mortality in breast cancer patients. We observed that our control group decreased fitness capacity by 24.5%, over double what has been reported previously in a meta-analysis [13]. Additionally, 4 years following completion of an exercise intervention during breast cancer treatment in 128 women, Witlox et al. observed that the standard of care control group had higher levels of fatigue and lower levels of physical activity compared to the intervention group [39]. Therefore, intervening during this treatment window may have durable effects in the long term.

Women recommended for neoadjuvant chemotherapy often have locally advanced tumors, HER2+ tumors, or TNBC [6]. As these tumor subtypes have the poorest prognosis in breast cancer patients, anxiety related to prognosis may significantly impact quality of life during treatment. While we primarily saw improvements in quality-of-life measures related to physical activity (physical function, impairment in daily activities, fatigue, leisure time activity), we also saw a trend for improvement in emotional limitations.

Our earlier studies indicated that breast cancer patients at the point of diagnosis have a strong desire for exercise programming following diagnosis [40]. However, we observed low acceptability (25.7%) in neoadjuvant chemotherapy patients compared to similar studies conducted in the United States [21, 38]. Therefore, we may have experienced selection bias due to the large percentage of patients who declined to participate. We also experienced a low accrual rate which necessitated opening the trial to additional sites. Eligibility criteria may also have influenced our low accrual rate (e.g. restrictions related to co-enrollment on other trials, and having a physical activity level above inclusion criteria). Depending on the primary aim of the exercise prescription in future trials, eligibility criteria (such as current physical activity level) can likely be discarded as exercise during treatment may improve efficacy of chemotherapy independent of current physical activity level. Some of the strengths of the study are also limitations. Many women cannot return to a center or clinic for supervised exercise training, yet, supervised exercise oncology interventions have demonstrated better adherence. While supervised exercise programs may have better adherence outcomes, [36] they are less feasible to deliver across sites or translate to implementation science [12]. Only about 12% of breast cancer diagnoses are recommended for neoadjuvant chemotherapy [41].This limited our sample size, which is a limitation, and also limits generalizability to other breast cancer patients.

## Conclusion

Our pilot study in neoadjuvant breast cancer patients was safe, improved quality of life, and demonstrated that approximately 65 min per week of home-based exercise is tolerable and effective in maintaining fitness capacity. Our remotely delivered exercise intervention utilized a phased approach to increase exercise intensity. It was difficult for patients to meet their tailored heart rate goals as both the intervention and their chemotherapy treatment course progressed. This indicates that it may be more efficacious to decrease the length of the introduction phase, and thus begin ramping patients to target intensities either as pre-habilitation or earlier in their treatment course prior to cumulative side effects of chemotherapy treatment. Larger trials may build on this pilot study and incorporate lessons learned. Ultimately, exercise training as an adjunctive therapy concomitant to chemotherapy may be utilized clinically for improved cancer outcomes. Potential implementation of exercise oncology in this regard will need to build on well-designed clinical trials which may find our feasibility study useful for such vertical translation.

## Data Availability

The datasets generated during and/or analyzed during the current study are available from the corresponding author on reasonable request.

## Abbreviations

ER+: Estrogen Receptor positive
PR+: Progesterone Receptor positive
HER2−: Human Epidermal Growth Factor Receptor 2 negative
HER2+: Human Epidermal Growth Factor Receptor 2 positive
TNBC: Triple Negative Breast Cancer
ACSM: American College of Sports Medicine
MET: Metabolic Equivalent
mL/kg/min: Milliliter per kilogram per minute
PSCI: Penn State Cancer Institute
PA: Pennsylvania
A&P: Andrews & Patel
UPenn: University of Pennsylvania
TCH+P: Taxotere: Carboplatin: Herceptin + Perjeta
ACT: Adriamycin: cyclophosphamide: Taxol
min/wk: Minutes per week
VO_2max_: Maximal oxygen consumption
COVID-19: Coronavirus disease of 2019
UT: Utah
HR_max_: Heart rate maximum
RPE: Rating of perceived exertion
WPAI: Work Productivity and Activity Impairment Questionnaire
EQ-5D: EuroQol 5D
SF-36: RAND 36-Item Short Form
MFSI-SF: Multidimensional Fatigue Symptom Inventory
DVD: Digital Video Disc
US: United States
Inc.: Incorporation
NY: New York
CONSORT: Consolidated Standards of Reporting Trials
CPET: Cardiopulmonary exercise test
